# Supplementary material for: Morphological and Genetic Heterogeneity in Aedes aegypti (Diptera: Culicidae) Populations Across Diverse Landscapes in West Africa
Source: Ecol Evol. 2025 Dec 17;15(12):e72748. doi: 10.1002/ece3.72748 (PMC12710532; doi:10.1002/ece3.72748)
Supplement: Supplementary file 2 — Figure S2: Heatmap showing the differences between wing shape and genetic distance. [file ECE3-15-e72748-s002.docx]

Figure S2: Heatmap showing the difference between wing shape and genetic distance.
