# Supplementary material for: Morphological and Genetic Heterogeneity in Aedes aegypti (Diptera: Culicidae) Populations Across Diverse Landscapes in West Africa
Source: Ecol Evol. 2025 Dec 17;15(12):e72748. doi: 10.1002/ece3.72748 (PMC12710532; doi:10.1002/ece3.72748)
Supplement: Supplementary file 3 — Table S1: Population information of sampled Aedes aegypti mosquitoes. [file ECE3-15-e72748-s004.docx]

Table S1: Population information of sampled *Aedes aegypti* mosquitoes

| **Country** | **Sampling**  **Period** | **Locality** | **Landscape type** | **Latitude** | **Longitude** | **Samples** | **Used for scaling pattern** | **Almost complete mitochondrial genome** | **Used for morphometric analysis (left/right wing)** |
| --- | --- | --- | --- | --- | --- | --- | --- | --- | --- |
| **Côte d’Ivoire** | **19.07.2022 - 25.07.2022** | **Bonoua** | **urban** | **5.26299** | **-3.60133** | **50** | **48** | **14** | **48/47** |
|  |  | **Samo** | **peri-urban** | **5.283013** | **-3.515762** | **50** | **48** | **15** | **48/48** |
|  |  | **Koffikro** | **rural** | **5.404234** | **-3.378681** | **50** | **50** | **15** | **50/49** |
|  |  | **Hévéa** | **sylvatic** | **5.415712** | **-3.49393** | **50** | **48** | **13** | **42/44** |
| **Burkina Faso** | **18.09.2021 - 23.09.2022** | **Ouagadougou** | **urban** | **12.3741667** | **-1.50028** | **75** | **61** | **10** | **67/66** |
|  |  | **Goundry** | **peri-urban** | **12.517910** | **-1.341019** | **50** | **30** | **1** | **41/40** |
|  |  | **Koassa** | **rural** | **12.005175** | **-1.330274** | **75** | **64** | **6** | **61/63** |
|  |  | **Niangoloko** | **sylvatic** | **10.26562** | **-4.91394** | **50** | **45** | **13** | **33/35** |
| **Ghana** | **05.10.2021 – 30.10.2021** | **Godokpoe** | **urban** | **6.605.838** | **0.496095** | **50** | **43** | **7** | **47/46** |
|  |  | **Lokoe-Site** | **peri-urban** | **6.586.111** | **0.437192** | **50** | **48** | **6** | **45/46** |
|  |  | **Kpenoe** | **rural** | **6.631.574** | **0.515839** | **50** | **48** | **0** | **47/47** |
|  |  | **Klefe** | **sylvatic** | **6.619.748** | **0.443849** | **50** | **31** | **3** | **28/30** |
| **Total** |  |  |  |  |  | **650** | **564** | **103** | **557/561** |
